# Strategic approach to optimisation of TB contact investigation in Nigeria

**DOI:** 10.5588/ijtld.22.0369

**Published:** 2023-02-01

**Authors:** O. Chukwuogo, B. Odume, C. Ogbudebe, S. Useni, N. Nwokoye, C. Dim, D. Nongo, R. Eneogu, T. Odusote, O. Oyelaran, E. Ubochioma, C. Anyaike, M. Gidado

**Affiliations:** 1 KNCV Tuberculosis Foundation Nigeria, Abuja, Nigeria; 2 Department of Obstetrics & Gynaecology, College of Medicine, University of Nigeria, Ituku-Ozalla, Nigeria; 3 United States Agency for International Development, Abuja, Nigeria; 4 National Tuberculosis and Leprosy Control Programme, Abuja, Nigeria; 5 KNCV Tuberculosis Foundation, Hague, The Netherlands

Dear Editor,

The COVID-19 pandemic has increased the global burden of TB.^1^ Nigeria is a high-burden country for TB, TB-HIV and multidrug-resistant TB (MDR-TB).^1^ In 2020, out of an estimated 452,000 incident TB cases in Nigeria, only 135,784 cases were notified.^2^ One of the strategies deployed by the National TB Programme (NTP) to find the missing TB cases is contact investigation (CI), which serves as an effective method for the timely diagnosis of TB patients and provision of TB preventive treatment (TPT) to eligible contacts.^3^ Although it is known that CI by NTPs will lead to earlier TB case detection and treatment,^4^ the practice in Nigeria has been largely passive and not client-centred. To improve the effectiveness of CI in Nigeria, the KNCV Tuberculosis Foundation introduced a client-centred approach through the USAID-funded TB LON Project. We present the results of this approach in this article.

From October 2020 to September 2021, KNCV Nigeria implemented active CI in 14 TB LON Project states (Bauchi, Kaduna, Katsina, Kano, Nasarawa, Plateau, Taraba, Anambra, Akwa Ibom, Benue, Cross River, Delta, Imo and Rivers). In each state, DOTS officers and health facility staff providing ad hoc TB screening were assessed and trained as contact investigators. The selected contact investigators were able to leverage the trust capital built with patients and were sensitive to cultural differences. Bacteriologically diagnosed pulmonary TB index cases (including childhood TB, MDR-TB and TB-HIV cases) were mapped, and their addresses compiled. The contact investigators visited the household of each assigned index case at a convenient time. Household contacts were identified, counselled and screened for TB.

The WHO-four-symptom screen (W4SS),^5^ comprising cough, fever, night sweats and weight loss, was conducted for each consenting contact. A spot sputum specimen was collected from each presumptive contact and transported in a cold box to an assigned diagnostic facility for same-day Xpert test. Anyone who could not produce sputum was linked, at no cost, to chest X-ray facilities for clinical diagnosis. Diagnosed TB patients were offered treatment. Asymptomatic contacts were linked to health facilities for TPT eligibility assessment, and consenting eligible contacts were placed on TPT as appropriate. We conducted a cross-sectional study using de-identified programme data on CI extracted from an electronic platform used to record project data (CommCare app). Data were exported to SPSS version 21 for descriptive and inferential data analyses. *P* < 0.05 was considered statistically significant. Ethics approval was not required for this study because de-identified pooled programme data were used. Verbal consent was sought from index TB patients before household visits and from contacts before screening. All diagnosed TB patients were offered treatment.

During the 1-year project, 28,354 TB index cases (males: 16,316, 57.5%; females: 12,038, 42.5%) had their contacts screened for TB. In all, 148,694 eligible contacts (males: 75,406, 50.7%; females: 73,288, 49.3%) were screened, giving an average of six screened contacts per index case. Out of all contacts screened, 45,258 (30.4%) presumptive TB cases were identified with a male to female proportion of 0.51 to 0.49, respectively (odds ratio [OR] 1.0, 95% confidence interval [CI] 0.97–1.02; *P* = 0.601) and modal age group for all contacts screened was 15–24 years. The details of the demographic characteristics of index cases, contacts and presumptive TB are shown in the Table. Of the 42,923 presumptive TB cases evaluated, 3,617 TB cases were identified, giving a TB prevalence of 8.4% among presumptive and 2.4% among the contacts screened. Prevalence of TB among male contacts was higher than among female contacts (2.7%, 2,001/75,406 vs. 2.2%, 1,616/73,288; OR 1.2, 95% CI 1.13–1.29; *P* < 0.001). For all contacts screened, the number needed to screen (NNS) was 42, while the number needed to test (NNT) was 12. The 25–34-years age group had the highest number of TB cases (*n* = 811, 22.4%). Childhood TB contributed 15.0% of all confirmed TB cases. Twenty-seven (0.7%) TB cases were drug-resistant, with a male to female ratio of 2:1. In all, 3,552 (98.2%) TB cases diagnosed were placed on treatment. Out of the eligible number, only 4,640 (29.0%) persons were placed on TPT.

**Table:** Demographic characteristics of index TB cases, screened contacts, presumptive TB and TB cases

Characteristic	Index TB cases *n* (%)	Contacts screened *n(*%)	Presumptive evaluated for TB *n* (%)	All TB cases *n* (%)	Drug-resistant TB *n* (%)	NNS	NNT
Total	28,354 (100.0)	148,694 (100.0)	42,923 (100.0)	3,617 (100.0)	27 (100.0)	42	12
Sex							
Male	16,316 (57.5)	75,406 (50.7)	21,411 (49.9)	2,001 (55.3)	18 (66.7)	38	11
Female	12,038 (42.5)	73,288 (49.3)	21,512 (50.1)	1,616 (44.7)	9 (33.3)	46	14
*P* value				<0.001			
Age group, years							
0–4	719 (2.5)	12,077 (8.1)	2,854 (6.6)	205 (5.7)	0 (0.0)		
5–14	1,659 (5.9)	22,937 (15.4)	5,699 (13.3)	336 (9.3)	1 (3.7)		
15–24	4,084 (14.4)	29,345 (19.7)	7,506 (17.5)	587 (16.2)	1 (3.7)		
25–34	6,163 (21.7)	26,442 (17.8)	7,654 (17.8)	811 (22.4)	6 (22.2)		
35–44	6,053 (21.3)	20,838 (14.0)	6,707 (15.6)	730 (20.2)	7 (25.9)		
45–54	4,524 (16.0)	16,515 (11.1)	5,359 (12.5)	465 (12.9)	7 (25.9)		
55–64	2,864 (10.1)	11,849 (8.0)	3,897 (9.1)	268 (7.4)	3 (11.1)		
≥65	2,288 (8.1)	8,691 (5.8)	3,247 (7.6)	215 (5.9)	2 (7.4)		

NNS = number needed to screen; NNT = number needed to test.

This one-year CI intervention was part of efforts to identify missing TB cases and improve TB case notification. Due to the large scope of the intervention, symptom screening was used to identify presumptive TB and diagnosis was done using Xpert test and chest radiography for clinical evaluation when indicated. The project findings support the known epidemiology of TB in Nigeria, characterised by male and 25–34 age group preponderance.^3^ The average number of contacts screened per index case in this project (*n* = 6) was good coverage compared to the average contact per index case (of about two) reported in a related study from Abia State, Nigeria.^6^ The 2.5% TB yield among contacts investigated in our study translates to a notification rate of 2,400/100,000 contacts in the implementation states, which is 11 times higher than Nigeria’s estimated TB incidence rate (219/100,000 population).^1^ This suggests a high TB burden among TB contacts, supporting the need to strengthen CI by the NTP. Given that active CI within the NTP had not attained universal coverage, the large pool of TB cases notified by this project would have remained undiagnosed. The low NNS on this project and local studies suggests that the risk of TB infection among household contacts is far higher than in other high TB transmission settings (such as the prisons). For example, a recent report from the largest prison in Kano State, Nigeria showed a higher NNS of 94 among inmates when compared to this project.^7^ Intuitively, the yield from CI is consistently higher than the estimated TB incidence and buttresses the effectiveness of CI as an active case-finding strategy.^6^,^8^–^10^ Major strengths of this project were that we succeeded in placing over 98% of TB cases diagnosed on appropriate treatment, and over a quarter of eligible household contacts on TPT. This was despite the prevailing challenge of getting apparently healthy individuals to accept TPT in our environment, which is attributable to a perception of low risk and vulnerability to a given condition (along with other community and health system factors).^11^–^14^ Future operational research should devise interventions to counter the impediments to achieving broader TPT coverage.^3^ Our study had some limitations due to CommCare data transcription; however, reference was made to data collected using paper-based tools to ensure completeness.

In conclusion, active CI yielded a high TB notification rate and demonstrated the possibility of integrating TPT within the CI programme. To reduce the number of missing TB cases in Nigeria, CI should become a mainstream aspect of routine TB care.
